# Can We and Should We Produce a model for Emergency Admissions derived from Primary Care Data?

**DOI:** 10.23889/ijpds.v1i1.295

**Published:** 2017-04-19

**Authors:** Kerry Bailey

**Affiliations:** Swansea University

## Objectives

Several Risk Prediction models exist for predicting emergency admissions. One exists derived solely from primary care data but this is only available in England. All the models currently available have limitations. The aim was to utilise the SAIL databank to develop a predictive model/tool using only primary care data that has comparable accuracy to the currently used risk stratification tools but overcomes the limitations of the current models available.

## Approach

A multidimensional approach was taken including literature reviews and qualitative interviews to the model to situate the statistical model. The need was identified to develop the contextual background in order to situate the statistical model development and understand the need for an improved model. Stakeholder groups were identified - patients, managers, General Practitioners and policy makers - and purposive sampling was used to obtain breadth and depth of representation into the subject area. Interviews were carried out in two stages initially to identify key themes and the breadth of the interest in stakeholder and influenced hypothesis generation. The second stage was to provide depth and clarity on specific themes with a more structured interview schedule. Results of the qualitave work triangulated with literature reviews influenced the statistical model build.

## Discussion

The statistical challenge of building Risk Prediction models well documented. Understanding the shortcoming of current models as perceived by stakeholders is necessary to produce a model of added value which performs statistically similar however has a different functionality. The qualitative work that was carried out to situate this model added value to the models development but also raised some interesting ethical issues. The validity and performance of the models although important is only one aspect that influences whether GPs would use a risk prediction model and how acceptable it is to stakeholders. None of the patients interviewed in this thought that their data was, or would be without their knowledge, used in risk prediction models yet all were patients at practices where this was happening or had morbidities which would have necessitated the use of several in primary care. 

